# Responsiveness and minimal clinically important difference of the EQ-5D-5L in cervical intraepithelial neoplasia: a longitudinal study

**DOI:** 10.1186/s12955-020-01578-8

**Published:** 2020-10-02

**Authors:** Xin Hu, Mingxia Jing, Mei Zhang, Ping Yang, Xiaolong Yan

**Affiliations:** 1 Department of Public Health, Shihezi University School of Medicine, Shihezi, Xinjiang China; 2 Department of Obstetrics and Gynaecology, The First Affiliated Hospital of Shihezi University School of Medicine, Shihezi, Xinjiang China

**Keywords:** Responsiveness, MCID, EQ-5D-5L, CIN, MDC

## Abstract

**Background:**

With the widespread clinical application of the five-level version of the EuroQol five-dimensional questionnaire (EQ-5D-5L), whether the questionnaire scores are responsive to changes in patients’ health and how much changes in questionnaire scores represent patients’ real health changes require consideration. Consequently, we assessed responsiveness and estimated the minimal clinically important difference (MCID) of the EQ-5D-5L in surgically treated patients with cervical intraepithelial neoplasia (CIN) to determine the relationship between MCID and minimal detectable change (MDC).

**Methods:**

We conducted a longitudinal, observational study. Participants were patients with CIN from the gynecology inpatient department of a grade-A tertiary hospital in Shihezi, Xinjiang, China. Participants completed the EQ-5D-5L and the Global Rating of Change Questionnaire (GRCQ) at baseline and one month post-surgery. The Wilcoxon signed-rank test was used to compare EQ-5D-5L scores pre- and post-treatment. We calculated the effect size (ES) and the standardized response mean (SRM) to quantitatively assess responsiveness. Distribution-based, anchor-based, and instrument-defined methods were used to estimate MCID. MCID to MDC ratios at individual- and group-levels were also calculated.

**Results:**

Fifty patients with CIN completed the follow-up investigation (mean age 44.76 ± 8.72 years; mean follow-up time 32.28 ± 1.43 days). The index value and EQ visual analogue scale (EQ VAS) of the EQ-5D-5L improved by 0.025 and 6.92 (all *p* < 0.05) at follow-up as compared to baseline respectively. The ES and the SRM of the index value were 0.47 and 0.42 respectively, indicating small responsiveness; while the ES and the SRM of EQ VAS were 0.50 and 0.49 respectively, indicating small to moderate responsiveness. The average (range) of MCIDs for index value and EQ VAS were 0.039 (0.023–0.064) and 5.35 (3.12–6.99) respectively. These values can only be used to determine whether patients have experienced clinically meaningful health improvements at the group level.

**Conclusions:**

The EQ-5D-5L has only small to moderate responsiveness in post-surgical patients with CIN, and the MCIDs developed in this study can be used for group-level health assessment. However, further study is needed concerning health changes at the individual level.

## Background

Cervical intraepithelial neoplasia (CIN) is a general term for cervical precancerous lesions, including low- and high-grade squamous intraepithelial lesions (HSIL) [1]. Studies have shown that the diagnosis of CIN negatively affects patients’ psychology [2, 3], and HSIL has a 31.3% probability of progressing to invasive cervical cancer in its natural state [4], which seriously endangers patients’ health. Favorably, surgical treatment is a definitive treatment for CIN, and the cure rate is very high [5]; thus, it is an effective measure to prevent the occurrence of invasive cervical cancer. Along with health-related quality of life (HRQoL), patient-reported outcomes (PROs) are clinical endpoints other than survival, such as the EuroQol five-dimensional questionnaire (EQ-5D) [6].

The EQ-5D is a simple, generic, and standardized instrument for HRQoL measurement [7]. At present, the questionnaire is widely used in the health assessment of the general population and patients with different diseases in China [8–11]. There are two versions of the EQ-5D. The three-level version (EQ-5D-3L) was first launched in 1990 [7]; however, owing to the obvious ceiling effect and its inability to sufficiently capture small changes [12–14], a five-level version (EQ-5D-5L) was developed in 2011 [15]. Recently, the Chinese version of the EQ-5D-5L has been released, and the value set based on the preferences of the Chinese population has been established [16].

In previous studies, clinical efficacy was generally judged based on the statistical differences in PROs; however, it could not indicate whether they were clinically significant [17]. The minimal clinically important difference (MCID), proposed by Jaeschke and colleagues [18], is the smallest difference in score in the domain of interest that patients perceive as beneficial and which would mandate, in the absence of troublesome side effects and excessive cost, a change in the patient’s management. MCID can help clinicians explain patients’ health changes implied by the change in the questionnaire score [19], and serves as an important indicator to judge the effectiveness of treatments from the patients’ point of view, which has clear implications for treatment measures in clinical practice.

Logically, MCID should be distinguished from the measurement error and therefore associated with the minimal detectable change (MDC). MDC represents the minimum change in the questionnaire scores required for real health changes and is mathematically related to the measurement error [20]. Through analyzing the relationship between MCID and MDC, we can further determine whether the established MCID is derived from patients’ real health change or the measurement error, which is crucial to judge changes in the health of the patient by applying the MCID in clinical settings. Moreover, the MCID and the MDC are related to responsiveness: the former is clinically oriented and focuses on the individual level [21], while the latter is based on the population.

Previous studies have confirmed the small to moderate responsiveness of the EQ-5D-5L in patients with pulmonary embolism [22], deep vein thrombosis [22], breast cancer [23], and those undergoing cataract surgery [24]. The MCID estimation of EQ-5D-5L has also been studied in different settings; however, the results vary. Specifically, the MCID of index value and the EQ visual analog scale (EQ VAS) in patients with chronic obstructive pulmonary disease undergoing pulmonary rehabilitation were 0.051 and 6.9 respectively [25], while the MCID of index value in patients with type 2 diabetes and elderly people with hypertension were 0.043 and 0.072 respectively [26, 27]. In addition, a study evaluating the relationship between MCID and MDC in patients undergoing hip or knee replacement showed that when the MCID of the index value was 0.32, it could be distinguished from measurement errors even at the individual level [28]. All these studies demonstrate the applicability of the EQ-5D-5L in clinical settings; however, to our knowledge, no studies have used the EQ-5D-5L to estimate the responsiveness and the MCID in surgically treated patients with CIN, nor have they analyzed the relationship between the MCID and the MDC.

The purposes of this study were (1) to evaluate the responsiveness of the EQ-5D-5L in patients with CIN who underwent surgery, (2) to estimate the MCID of the EQ-5D-5L, and (3) to analyze the relationship between MCID and MDC.

## Methods

### Participants and investigation process

This was a longitudinal, observational study. Participants were recruited from the gynecology inpatient department of a grade-A tertiary hospital in Shihezi, Xinjiang, China between November 2018 and August 2019. Inclusion criteria were (1) a positive cervical tissue biopsy result diagnosis of CIN as determined by a professional gynecologist as the primary admission diagnosis for the first time; (2) aged > 18 years; (3) Han ethnicity; (4) untreated before the baseline investigation; (5) the ability to express inner feelings clearly; (6) no severe comorbidities, mental illness, or cognitive impairment; and (7) willing to participate in this study. Exclusion criteria were (1) no CIN-related surgical treatment during hospitalization and (2) the establishment of invasive cervical cancer as the pathological diagnosis upon discharge.

The baseline investigation was conducted through face-to-face interviews with patients when they were admitted, and the follow-up visit was performed one month after the surgery by telephone. The same investigator was responsible for both surveys. Investigators were postgraduates with a medical background and had been trained professionally.

### Measurement

#### Demographic and medical characteristics

Age, marital status, education level, body mass index (BMI), medical insurance, and household income were obtained through face-to-face interviews with patients. The disease duration, histopathological results, and surgical approach were collected through electronic medical records.

#### EQ-5D-5L and Global Rating of Change Questionnaire (GRCQ)

The EQ-5D-5L consists of a short descriptive system and the EQ VAS. The descriptive system comprises five dimensions, each describing a different aspect of health: mobility, self-care, usual activities, pain/discomfort, and anxiety/depression. Each dimension has five response levels of severity: *no problems*, *slight problems*, *moderate problems*, *severe problems*, and *unable to/extreme problems* [29]. After responding to each dimension, a dimension score is obtained, which is defined as the parameter score corresponding to the patients’ response to the severity level in each dimension [16]. The larger the score, the more serious the problem. Further, a five-digit code can be summarized to describe the state of the individual’s health, which can be converted into a single number–index value. In China, the value ranges from − 0.391 to 1.000, and scores on the ends represent “the worst health state” and “the best health state” respectively [16, 29]. EQ VAS is a vertical scale concerning overall health quantity: 0 and 100 are located at the poles, which represent “the worst health you can imagine” and “the best health you can imagine” respectively [29]. The higher the index value and EQ VAS, the better individual’s health was deemed to be.

The GRCQ is an external anchor for determining the MCID of questionnaire scores, which contains only one question [18]: “How does your overall health change after treatment?” Transition ratings are based on a 5-point Likert scale: “much better,” “a little better,” “about the same,” “a little worse,” and “much worse.”

### Statistical analyses

Participants’ characteristics were described by mean ± standard deviation (SD) and numbers and percentages (%). Comparisons of baseline and follow-up scores of the EQ-5D-5L were made using the Wilcoxon signed-rank test.

#### Responsiveness

Effect size (ES) and standardized response mean (SRM) were used to evaluate responsiveness, which were classified as per Cohen’s d standard [30]: < 0.2, no responsiveness; 0.2 to 0.49, small; 0.5 to 0.79, moderate; and ≥ 0.8, large.

#### Minimal clinically important difference

There is still no consensus on the best method for estimating MCID [31]; however, distribution-based and anchor-based methods are commonly used [32, 33], and the latter is preferred [34]. In addition to the above two methods, we also adopted the instrument-defined method, which is only relevant for preference-based measurements such as the EQ-5D-5L, and the MCID estimation is completed based on the simulated transition of health states [34]. All three methods have their own merits and limitations (see Table 1 for details).

**Table 1:** Advantages and limitations of distribution-based, anchor-based, and instrument-defined methods for MCID calculation

Method	Advantages	Limitations
Distribution-based [17, 32, 35]	Considering measurement precision Clear formula, easy to implement	Based on statistical distributions of data and the reliability of the instrument, so that the MCID would be affected by the sample and the measurement characteristics of instrument itself Several different values may be obtained based on different calculation formulas Not based on changes in patient-reported results and therefore does not provide a good indication of the importance of the observed changes
Anchor-based [17, 32, 35, 36]	Define “minimal importance” explicitly and incorporate it into these methods Can provide MCID with clinically significant explanations	Anchor question may not fully capture changes in the PROs that may reflect more than one type of outcome MCID depends on what transition rating on the anchor question is considered as “clinically important” Does not consider measurement precision Recall bias
Instrument-defined [34]	A simple method that can be easily applied by other researchers to calculate the MCIDs for the studied instruments using scoring algorithms for other populations Using several health transitions as reference points or standards for minimally important change, resulting in MCID based on multiple internal anchors Does not require collection of primary data; thus, it is resource- and time- saving	Some instrument-defined health transitions may not occur in reality, which may lead to biased estimates Some health transitions used may represent trivial or large changes that may lead to biased estimates Some “smallest” health transitions may represent changes that are larger than the MCID

In the distribution-based method, 0.5SD and 1 standard error of measurement (SEM) for MCID is calculated as follows: $$SEM = SD \times \sqrt {{1 - }r_{\begin{subarray}{l} {\text{test } - \text{ retest}} \\ \end{subarray} } }$$ [17]. Based on previous study, the test–retest reliability was equal to 0.82 [37]. The anchor-based method used the GRCQ as an external anchor and regarded the transition rating corresponding to “a little” changes as the MCID. Since no patient responded to the anchor question as “worse” in this study, the MCID estimate was performed only for the transition rating “better.” Therefore, the MCID was defined as the difference of the mean change scores of the EQ-5D-5L between the transition ratings of GRCQ that were “a little better” and “about the same” [38, 39].

The instrument-defined method is based on the average of index value differences in the descriptive system of the EQ-5D-5L between the baseline health state and single-level transitions to other health states [26]. MCID estimates can be classified into three categories according to the direction of single-level transitions of baseline health states: only transitions to a better state, only transitions to a worse off state, and all single-level transitions [26]. This study only used the first category. If the baseline health state was “11111,” we excluded it from the MCID estimate because it could no longer be improved [26]. In addition, the maximum-valued scoring parameter in the Chinese value set, the conversion parameter between “moderate problems” and “severe problems,” was excluded from MCID estimate based on the instrument-defined method. The reason is that the conversion parameter among these two levels exceed other adjacent levels at least 1.39 times in all five dimensions, and potentially risks overestimating the MCID [40]. The calculation method of the MCID based on an instrument-defined method is detailed elsewhere [34, 40].

#### Minimal detectable change

At the 95% confidence level, $MDC = SEM \times \sqrt 2 \times 1.96$, MDC_95%(ind)_ measures the smallest detectable change of scores that are beyond the measurement error, at the individual level [41]. According to de Boer and colleagues’ methodology, the MDC in a group of people, MDC_95%(group)_, is equal to MDC_95%(ind)_ divided by $\sqrt n$, where n is the sample size [42]. Ratios of MCID to MDC_95%(ind)_ and MDC_95%(group)_ were calculated to illustrate the relationship between MCID and MDC [42]. If the ratio is greater than 1, the MCID can be distinguished from the measurement error and used to determine the health changes at the individual- or group- levels [42].

SPSS (version 24.0) and R studio were used for statistical analyses, and *p* < 0.05 was considered significant.

## Results

**Fig. 1:**
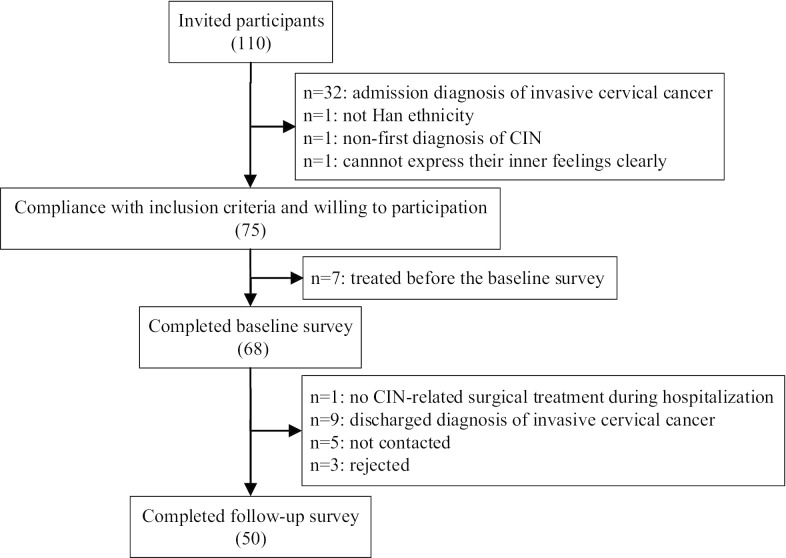
Flow chart of participants recruitment and follow-up

A total of 110 patients were invited to participate in the study, of which 68 met the inclusion criteria and accomplished the baseline investigation. Fifty (73.53%) patients completed the follow-up visit on average of 32.28 ± 1.43 days after surgery. The reasons for non-completion were “no CIN-related surgical treatment during hospitalization” (n = 1), “discharged diagnosis of cervical invasive cancer” (n = 9), “not contacted” (n = 5), and “rejected” (n = 3; Fig. 1).

The average age and disease duration of patients who completed the follow-up survey was 44.76 ± 8.72 years and 0.66 ± 0.92 months. Most patients were married (92.00%), had at least a junior school education (88.00%), had a BMI within the normal range (52.00%), and had medical insurance (98.00%); however, only 4.00% of patients had a moderate household income. Nearly all of the patients (94.00%) had HSIL, of which carcinoma in situ accounted for 23.40%, and cervical cone resection was the main surgical approach (98.00%; Table 2). With regard to the GRCQ transition ratings, 16 patients were “much better,” 10 were “a little better,” 24 were “about the same,” and there was no response to “worse.”

**Table 2:** Demographic and medical characteristics of CIN patients

Characteristics	n	%
Age, years (mean ± SD)	44.76 ± 8.72	
Marital status		
Married	46	92.00
Other	4	8.00
Education level		
Primary school and below	6	12.00
Junior school	18	36.00
Senior school	9	18.00
University and above	17	34.00
BMI, kg/m^2^		
< 18.5	2	4.00
18.5–24	26	52.00
24–28	16	32.00
≥ 28	6	12.00
Medical insurance		
Yes	49	98.00
No	1	2.00
Household income, yuan		
≤ 30,000	5	10.00
30,000–80,000	23	46.00
80,000–150,000	20	40.00
> 150,000	2	4.00
Disease duration, month (mean ± SD)	0.66 ± 0.92	
Histopathology		
CIN1	3	6.00
CIN2	14	28.00
CIN3	22	44.00
Carcinoma in situ	11	22.00
Surgical approach		
Cervical cone resection	49	98.00
Total hysterectomy	1	2.00

### Responsiveness of the EQ-5D-5L

The results demonstrated that scores of self-care and usual activities did not change before and after treatment, while scores of mobility, pain/discomfort and anxiety/depression decreased by 0.003 (*p* = 0.317), 0.004 (*p* = 0.405), and 0.018 (*p* = 0.010), respectively, which indicated an improvement of these dimensions at follow-up (Table 3).

**Table 3:** Comparison of scores before and after treatment in each dimension of descriptive system

Dimensions	Baseline	Follow-up	Difference	*p* value
Mobility	0.003 ± 0.022	0.000 ± 0.000	− 0.003 ± 0.022	0.317
Self-care	0.000 ± 0.000	0.000 ± 0.000	0.000 ± 0.000	1.000
Usual activities	0.000 ± 0.000	0.000 ± 0.000	0.000 ± 0.000	1.000
Pain/discomfort	0.010 ± 0.027	0.006 ± 0.018	− 0.004 ± 0.032	0.405
Anxiety/depression	0.027 ± 0.039	0.010 ± 0.027	− 0.018 ± 0.046	0.010

Among all patients, index value and EQ VAS increased by 0.025 and 6.92 (all *p* < 0.05) after treatment, respectively. The ES and the SRM of index value were 0.47 and 0.42 respectively, indicating a small responsiveness; and the ES and SRM of EQ VAS were 0.50 and 0.49 respectively, indicating small to moderate responsiveness. In patients who responded to the question on GRCQ transition rating as “improvement” (including “a little better” and “much better”), the index value change was positive (△index value = 0.039, *p* = 0.004); i.e., ameliorating the HRQoL. ES and SRM were 0.59 and 0.67 respectively, suggesting a moderate effect. EQ VAS presented similar results as index value. At follow-up, EQ VAS exceeded baseline 9.27 (*p* = 0.001) on average, and it had moderate responsiveness (ES = 0.70, SRM = 0.71). As for patients who were “about the same,” the change in index value and EQ VAS were 0.010 and 4.37 respectively; however, these were non-significant differences (all *p* > 0.05), and both were small even no responsiveness (index value: ES = 0.29, SRM = 0.17; EQ VAS: ES = 0.29, SRM = 0.30; Table 4).

**Table 4:** Responsive to GRCQ of the EQ-5D-5L at 1 month and comparison of scores before and after treatment

Variables	Index value			EQ VAS		
	All (n = 50)	Improvement (n = 26)	About the same (n = 24)	All (n = 50)	Improvement (n = 26)	About the same (n = 24)
Baseline score	0.960 ± 0.053	0.953 ± 0.066	0.967 ± 0.035	83.80 ± 13.98	83.65 ± 13.16	83.96 ± 15.11
Follow-up score	0.985 ± 0.034	0.992 ± 0.020	0.977 ± 0.044	90.72 ± 8.70	92.92 ± 7.39	88.33 ± 9.52
Score change	0.025 ± 0.060	0.039 ± 0.058	0.010 ± 0.059	6.92 ± 14.01	9.27 ± 13.13	4.37 ± 14.77
*p* value	0.034	0.004	0.774	0.001	0.001	0.150
ES	0.47	0.59	0.29	0.50	0.70	0.29
SRM	0.42	0.67	0.17	0.49	0.71	0.30

### Estimation of MCID and MDC

**Fig. 2:**
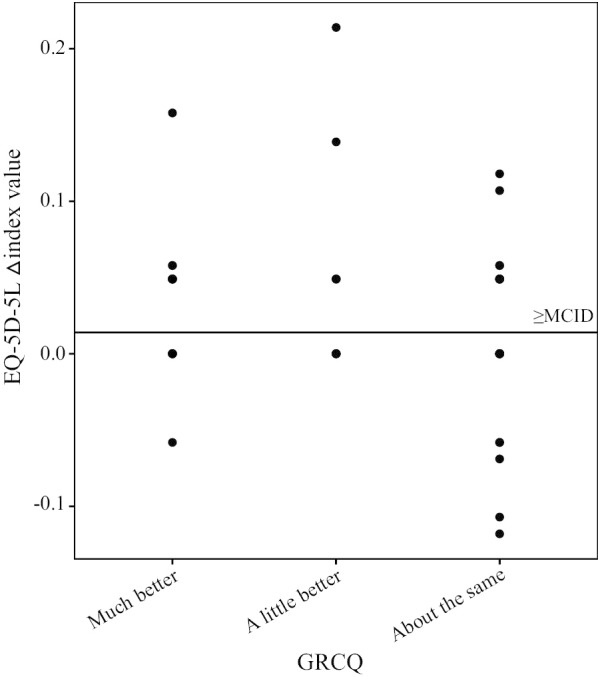
Scatter plot of changes in index value and GRCQ transition ratings. *Note.* The horizontal solid line represents the MCID of index value obtained by the anchor-based method

**Fig. 3:**
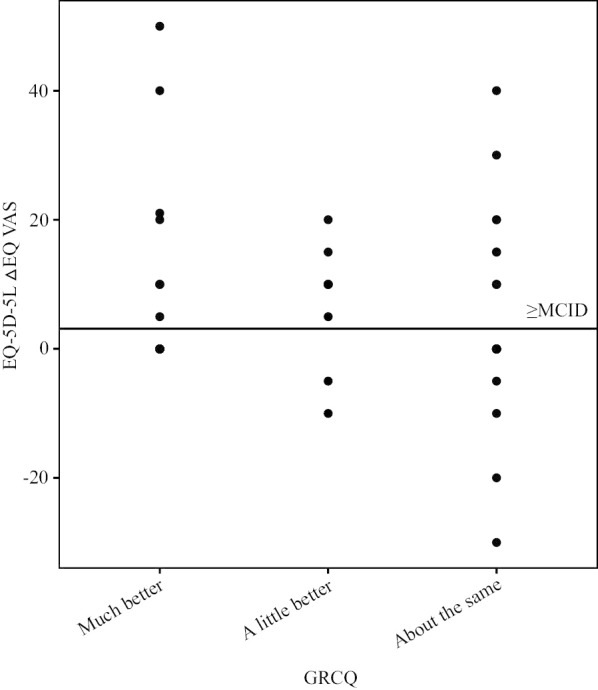
Scatter plot of changes in EQ VAS and GRCQ transition ratings. *Note.* The horizontal solid line represents the MCID of EQ VAS obtained by the anchor-based method

Table 5 displays MCIDs estimated by three methods. The MCID range of index value obtained by the distribution-based method was 0.023 to 0.027, and the MCID range of EQ VAS was 5.93 to 6.99. The result of MCID estimated by the anchor-based method had an index value of 0.041 and an EQ VAS of 3.12. The MCID of index value based on the instrument-defined method was 0.064. Figures 2 and 3 show the scatter plot of EQ-5D-5L score change in accordance with the transition rating of GRCQ. As shown, among the patients with a transition rating of “improvement,” the △index value of 14 patients and △EQ VAS of 16 patients were not less than the MCID, accounted for 53.85% and 61.54% respectively.

**Table 5:** MCIDs of the EQ-5D-5L estimated through three methods and the relation to the MDC at the individual and group levels

Variables	Index value				EQ VAS		
	0.5SD	1SEM	Anchor-based method	Instrument-defined method	0.5SD	1SEM	Anchor-based method
MCID	0.027	0.023	0.041	0.064	6.99	5.93	3.12
MDC_95%_							
Ind	0.064				16.44		
Group	0.009				2.32		
Ratio							
Ind	0.42	0.36	0.64	1.00	0.43	0.36	0.19
Group	3.00	2.56	4.56	7.11	3.01	2.55	1.24

Table 5 also shows ratios of MCID to MDC_95%(ind)_ and MDC_95%(group)_. The index value and EQ VAS have MDC_95%(ind)_ of 0.064 and 16.44, and MDC_95%(group)_ of 0.009 and 2.32. The ratios of MCID to MDC_95%(ind)_ of index value and EQ VAS were all < 1. This illustrated that MCID cannot discriminate the score change of the EQ-5D-5L from the measurement error at the individual level. Nevertheless, the ratios of MCID to MDC_95%(group)_ for index value and EQ VAS exceeded 1, symbolizing that we have 95% confidence that the 50 patients in this study experienced the smallest significant improvement, and surgical treatment could be used as an effective treatment for patients with CIN at the group level. The ratios highlight the fact that the instrument-defined method and the distribution-based method have relatively good performances in index value and EQ VAS; therefore, these two methods were recommended for the MCID estimation of the EQ-5D-5L in post-surgical patients with CIN.

## Discussion

This longitudinal study of patients with CIN showed that the EQ-5D-5L was responsive to change in health after surgery, and the effect size was between small and moderate. The index value and EQ VAS after treatment were on average 0.039 and 5.35, which can be considered an improvement in health from patients’ perspective. However, the MCID estimated in this study can only represent truly meaningful change of the HRQoL score at the group levels, not the individual levels.

Among all dimensions, the anxiety/depression dimension was the most improved post-surgery, and the only one with a significant score change. This is similar to the results of a longitudinal HRQoL assessment of patients with CIN by Xie et al., who assessed patients one month after treatment, and found that the average improvement in mental component summary scores (MCS) measured by the SF-36 questionnaire was higher than that of the physical component summary scores (PCS; △MCS:7.05 vs. △PCS:1.47) [43]. A possible explanation is that, in general, CIN does not produce symptoms or signs that affect patients’ ability to perform, whereas a CIN diagnosis has a negative psychological impact [2, 3]. Howbeit, the psychological support of doctors, good prognosis examples of patients, and increased awareness of disease may ameliorate the psychological impact.

In all patients, the positive changes in the index value and EQ VAS also coincided with other studies. A prospective study of Chinese patients with CIN conducted by Zhao et al. found that EQ-5D scores 1 month after treatment were significantly better than at baseline [6]. Therefore, we considered that post-surgical changes to patients’ health can be qualitatively judged by the change in the EQ-5D-5L score. Interestingly, the index value and EQ VAS of patients whose response to the GRCQ was “improvement” increased significantly, while a different result was discovered among those who responded “about the same.” Bilbao et al. revealed similar results among patients who underwent surgery for hip or knee osteoarthritis. They observed that the mean change of the EQ-5D-5L score was positive in “improved” group [28]. Patients’ perceived health changes, as measured by the GRCQ, were consistent with EQ-5D-5L score changes, even though the GRCQ has only one question and the EQ-5D-5L is a multi-dimensional, multi-attribute questionnaire. Thus, the GRCQ is a simple and credible choice for determining whether health changes occurred when multiple-items questionnaires cannot be used.

Two of the most commonly used indicators of responsiveness—ES and SRM—were used to estimate the degree of change in patients’ health [44–46]. The effect size of the EQ-5D-5L across the entire sample was only between small and moderate, which mirrored previous studies. Chen et al. assessed the responsiveness of the EQ-5D-5L with 65 Taiwanese patients who were receiving rehabilitation after a stroke, and discovered that the effect sizes ranged from 0.40 to 0.63 for the index value and 0.30 to 0.34 for the EQ VAS—suggesting small to moderate responsiveness [47]. Furthermore, the effect size of index value was only 0.20 in patients after cataract surgery [48]. Another study of obese patients showed that the index value and the EQ VAS had only small responsiveness after bariatric surgery [49]. These findings suggest that the EQ-5D-5L is responsive to various conditions, which clarifies that health changes were clinically relevant rather than random errors; nonetheless, the small responsiveness is noteworthy. The reason may be that the study population had chronic diseases, and experienced a slow deterioration of their health and had a weak perception of the change in their health as compared to patients with acute disease who may recover rapidly.

Some researchers believe that responsiveness may depend on the direction of changes in health state and the individuals’ health state at baseline [36]. The current results do give credence to the theory. We found a moderate responsiveness to the index value and the EQ VAS in patients with improved health states, while small or no responsiveness was found in patients with no change. In addition, the baseline scores of index value and EQ VAS in “improvement” surgical patients were lower than those that were “about the same,” while the change in the score was higher in the former than the latter. Statistically, the responsiveness of patients with improved health states must be better than that of “about the same” patients.

Responsiveness of the EQ-5D-5L in patients with improved health states was also studied in other populations; however, the results were inconsistent. In patients with acute asthma who underwent one month of treatment and self-reported improved health states, the index value had moderate to large responsiveness with the effect size ranged from 0.63 to 0.95 [50]. Golicki et al. revealed that the EQ-5D-5L was consistently responsive in patients who had a stroke, who displayed improved health four months after treatment: the index value showed a moderate ES (0.51–0.71) and a moderate to large SRM (0.69–0.86), while the ES of EQ VAS ranged from 0.51 to 0.65 and the SRM ranged from 0.59 to 0.69 [51]. Another study of patients with osteoarthritis six months after surgery showed that patients with improved health states had an ES and SRM of 1.48 for index value, and an ES of 0.82 and SRM of 0.90 for EQ VAS [28]. Through the above, we found that although the responsiveness of “improvement” patients was at least moderate, the effect size of each study was quite different. The source of the difference may be attributed to the participants’ unique characteristics or the different time intervals between the two measurements [47]. Because longer time intervals allow for sufficient time to respond to one’s physical condition, it is reflected in larger score changes, resulting in a larger effect size to reflect the degree of change in health conditions upon full recovery, and vice versa [37].

MCID is a vital component of the questionnaire application. Previous studies have utilized the mean change of MCID scores in the anchor-based method [52, 53]; however, this does not consider the possible impact of HRQoL scores over time in patients who reported no health changes during follow-up [39]. However, in this study, the absolute value of score change in participants that scored “a little better” minus the score change in participants that scored “about the same” was used as the MCID; thus, we eliminated the potential impact of time on the MCID estimation.

Besides the distribution-based and anchor-based methods, the instrument-defined method can also be used to triangulate the MCID. Luo et al. used the instrument-defined method to estimate the MCID for the EQ-5D-3L, and the result was parallel to the published estimate; therefore, the instrument-defined method was regarded as an effective method for MCID estimation [34]. Owing to our results, we deem that the instrument-defined method can be used for the MCID estimation of the EQ-5D-5L in patients with CIN.

Concerning the relationship between MCID and MDC, the results demonstrated that the MCID estimated for index value and EQ VAS by the three methods can, at the group level, explain that the score change was a result of health changes rather than measurement error. However, MCID of index value and EQ VAS both cannot account for individual health changes at the 95% confidence level, possibly because of the inclusion of patients with different histopathological histories. In this study, the proportion of patients with carcinoma in situ was 22.00%. Although this belongs to CIN [1], compared with other pathological grades, it involves a higher risk of progressing to invasive cancer [54], and patients had lower psychological expectations of health changes; therefore, the result may be a result of the different criteria that patients use to judge their health changes. Another possible explanation may be that, although we only included first-diagnosed patients, the HRQoL scores at baseline of some patients with a longer disease duration may have improved compared to those more recently diagnosed, resulting in the baseline score of the entire sample being raised. Therefore, the possibility of underestimating MCID leads to it being less than MDC_95%(ind)_. The current results should be further validated in patients with the same pathological grade and the same disease duration.

This study had several advantages. First, we used a combination of qualitative and quantitative approaches to assess responsiveness, which increases the credibility of the results. Second, in addition to the distribution-based and anchor-based methods, using the instrument-defined method for MCID estimation highlights the value of our results. Third, we analyzed whether the MCID estimated by each method can reflect true health changes at individual and group levels, which allowed us to determine the reliability of MCID and avoid the incorrect application or interpretation of the MCID. Although judging whether MCID differs from the measurement error is a logical next step after MCID estimation [42], only a few studies have done this [55, 56]. Finally, there was no investigator-based measurement bias because both time-point surveys for each patient were performed by the same investigator.

This study also had several limitations. Apart from the GRCQ, a disease-specific questionnaire was a commonly used anchor in previous studies [25, 38]; however, we did not use a disease-specific questionnaire for CIN such as the Functional Assessment of Chronic Illness Therapy–Cervical Dysplasia, since there is no Chinese version [57]. Although the GRCQ has only one question, it is the accepted anchor for MCID estimation at this stage [17]. Studies have shown that, if health state changes in different directions, the MCID may also be different [58]. Because no patients reported a worsen change in their health condition in this study, MCID could not be estimated for this group of patients. Future studies could develop the MCID for such patients to determine whether it differs from improved patients. It is well known that MCID changes are associated with demographic characteristics, interventions, etc.[33, 59]; therefore, the current results cannot be generalized to other clinical settings. Another limitation is that different interview methods used during baseline and follow-up surveys may lead to an information bias. Furthermore, the small sample size may affect MCID accuracy, although, this study met the basic requirements for MCID estimation [60].

## Conclusion

The EQ-5D-5L was responsive to surgically treated patients with CIN but with a small to moderate effect size. The results yielded an index value of 0.039 and an EQ VAS of 5.35. The analysis of the relationship between MCID and MDC revealed that the MCID developed for index value and EQ VAS can only determine whether patients actually experienced meaningful health changes at the group level; therefore, further study is needed concerning changes at the individual level.

## Data Availability

The datasets used and/or analysed during the current study are available from the corresponding author on reasonable request.

## Abbreviations

CIN: Cervical intraepithelial neoplasia
HSIL: High-grade squamous intraepithelial lesion
HRQoL: Health-related quality of life
PROs: Patient-reported outcomes
EQ-5D: EuroQol five-dimensional questionnaire
EQ-5D-3L: The three-level version of the EuroQol five-dimensional questionnaire
EQ-5D-5L: The five-level version of the EuroQol five-dimensional questionnaire
MCID: Minimal clinically important difference
MDC: Minimal detectable change
EQ VAS: EQ visual analogue scale
BMI: Body mass index
GRCQ: Global Rating of Change Questionnaire
SD: Standard deviation
ES: Effect size
SRM: Standardized response mean
SEM: Standard error of measurement
MCS: Mental component summary scores
PCS: Physical component summary scores
